# Long-term treatment with anti-CD20 monoclonal antibodies is untenable because of risk: Commentary

**DOI:** 10.1177/13524585221101138

**Published:** 2022-06-09

**Authors:** Jan Lycke, Anders Svenningsson

**Affiliations:** Department of Clinical Neuroscience, Institute of Neuroscience and Physiology, The Sahlgrenska Academy, University of Gothenburg, Gothenburg, Sweden; Department of Clinical Sciences and Department of Neurology, Danderyd Hospital, Karolinska Institutet, Stockholm, Sweden

The crucial role of B-cells in the immunopathogenesis of multiple sclerosis (MS) was revealed in the first trials of monoclonal antibodies (mAbs) targeting CD20 on B-cells.^1,2^ Since then, accumulating evidence confirms that the three major anti-CD20 therapies, rituximab, ocrelizumab, and ofatumumab, are all highly effective, reducing relapse rate and lesion formation on magnetic resonance imaging (MRI). Due to the low passage of mAbs over the blood–brain barrier, the mechanism of action from anti-CD20 therapies is mainly in the periphery, giving rise to undetectable levels of B-cells in blood. Nevertheless, in addition to a strong anti-inflammatory effect, an effect on degeneration is also obtained, halting disability worsening and the rate of brain and spinal cord atrophy. This effect is probably secondary to immunosuppression, and trials of rituximab and ocrelizumab in primary progressive MS suggest that lower progression rate is mainly achieved in patients with signs of inflammatory activity. However, safety of anti-CD20 therapies has been a concern in pivotal trials, their open label extensions, as well as in observational studies, in particular the risk of serious infections.^3^ This risk has also received attention during the SARS-CoV-2 (severe acute respiratory syndrome coronavirus 2) pandemic where several reports unanimously confirm an increased risk for severe COVID-19 (coronavirus disease 2019) infection^4^ and a lower IgG antibody response after vaccination in rituximab- and ocrelizumab-treated patients.^5^ This raises important critical questions on the selection of patients, monitoring safety and possible strategies to reduce risks of serious infection and improve humoral vaccine response during anti-CD20 treatment.

Dr Zecca and Dr Gobbi argue that long-term anti-CD20 mAb therapies are untenable because of the progressively increased risk of adverse event burden, including serious and opportunistic infections, malignancies, and impaired immune response to vaccines.^6^ To mitigate the risk of serious adverse events, they suggest anti-CD20 as an induction therapy or modified anti-CD20 dosing schedules. In contrast, Dr Tallantyre considers that the benefits of long-term anti-CD20 outweigh the risks.^7^ The risk of secondary IgG deficiency is limited, and although the antibody response to vaccination is impaired during anti-CD20 therapy, the T-cell response is maintained,^8^ which should reduce the risk for severe infection. In patients with hypogammaglobulinemia, Tallantyre suggests the use of serology surveillance, prophylactic antibiotics, or immunoglobulin substitution.

There seem to be two major strategies to mitigate the risk with anti-CD20 therapies in MS. First, a selection of patients, suggested by both the Yes and No positions, should improve safety. Thus, older age and age-related factors including severe disability, immunosenescence, comorbidities, and use of other immunosuppressive therapies should limit the use of anti-CD20 treatment. The second strategy is the adaptation of the anti-CD20 dosing schedule. High efficacy of rituximab treatment in MS seems to be maintained with a comparable low dose^9^ and extended dosing interval or even discontinuation did not increase the risk of reappearance of disease activity in a retrospective study.^10^ Thus, improved safety of anti-CD20 treatment should be achievable. A major advantage with anti-CD20 therapies is the possibility to monitor B-cell counts and IgG levels in blood and thereby allowing modifications of dosing schedules to reduce risks and improve humoral response after vaccination.
